# Low-cost FDM 3D-printed modular electrospray/electrospinning setup for biomedical applications

**DOI:** 10.1186/s41205-020-00060-x

**Published:** 2020-04-14

**Authors:** Jing Huang, Vasileios Koutsos, Norbert Radacsi

**Affiliations:** School of Engineering, Institute for Materials and Processes, The University of Edinburgh, Robert Stevenson Road, Edinburgh, EH9 3FB UK

**Keywords:** 3D printing, Low-cost, Electrospinning, Electrospray

## Abstract

Here, we report on the inexpensive fabrication of an electrospray/electrospinning setup by fused deposition modelling (FDM) 3D printing and provide the files and parameters needed to print this versatile device. Both electrospray and electrospinning technologies are widely used for pharmaceutical, healthcare and bioengineering applications. The setup was designed to be modular, thus its parts can be exchanged easily. The design provides a safe setup, ensuring that the users are not exposed to the high voltage parts of the setup. PLA, PVA, and a thermoplastic elastomer filament were used for the 3D printing. The filament cost was $100 USD and the rig was printed in 6 days. An Ultimaker 3 FDM 3D printer was used with dual print heads, and the PVA was used as a water-soluble support structure. The end part of the setup had several gas channels, allowing a uniform gas flowing against the direction of the nanoparticles/nanofibers, enhancing the drying process by enhancing the evaporation rate. The setup was tested in both electrospray and electrospinning modes successfully. Both the .sldprt and .stl files are provided for free download.

## Introduction

Nanotechnology has emerged as a state-of-the-art tool for biomedical applications and has attracted biotechnology, pharmaceutical, and healthcare industries during recent decades [1]. Electrohydrodynamic atomization (EHDA) is a popular technique for producing nano-sized objects by applying high voltage for applications in the biomedical field. Both electrospray ionization deposition and electrospinning techniques are based on the EHDA technique.

The electrospray technique (also called electrospray ionization deposition) is one of the most efficient ways for the preparation of nanospheres and nanoparticles as it is a simple and inexpensive method [1–3]. Electrosprayed nanoparticles are often used for pharmaceutical, biological or medicinal applications [4–6]. For example, electrospray can be used to fabricate nanoparticles loaded with drugs for nanoparticle drug delivery or loaded with cell growth factors for tissue engineering [4, 7, 8].

Electrospinning is a widely used method for pharmaceutical, medicinal or biological applications [9–12] as it can process solutions, melts, or even suspensions into long nano/micro-fibers [13]. It is the only technique for scaling up continuous nanofiber production [14]. Electrospinning is a modern technique in medicine that can fabricate nanostructures, which are mimicking our body’s extracellular matrix by the high surface area, providing an excellent scaffold for cell attachment [15]. This makes electrospinning an attractive technique for tissue engineering applications, including vascular graft fabrication [16, 17]. It is also widely used in medical diagnosis and drug delivery as they can immobilize the recognition element or active pharmaceutical ingredient due to the large surface area and porosity [15, 18, 19]. Recently, electrospinning has been also used for replica molding and producing three-dimensional scaffolds [20, 21].

**Fig. 1:**
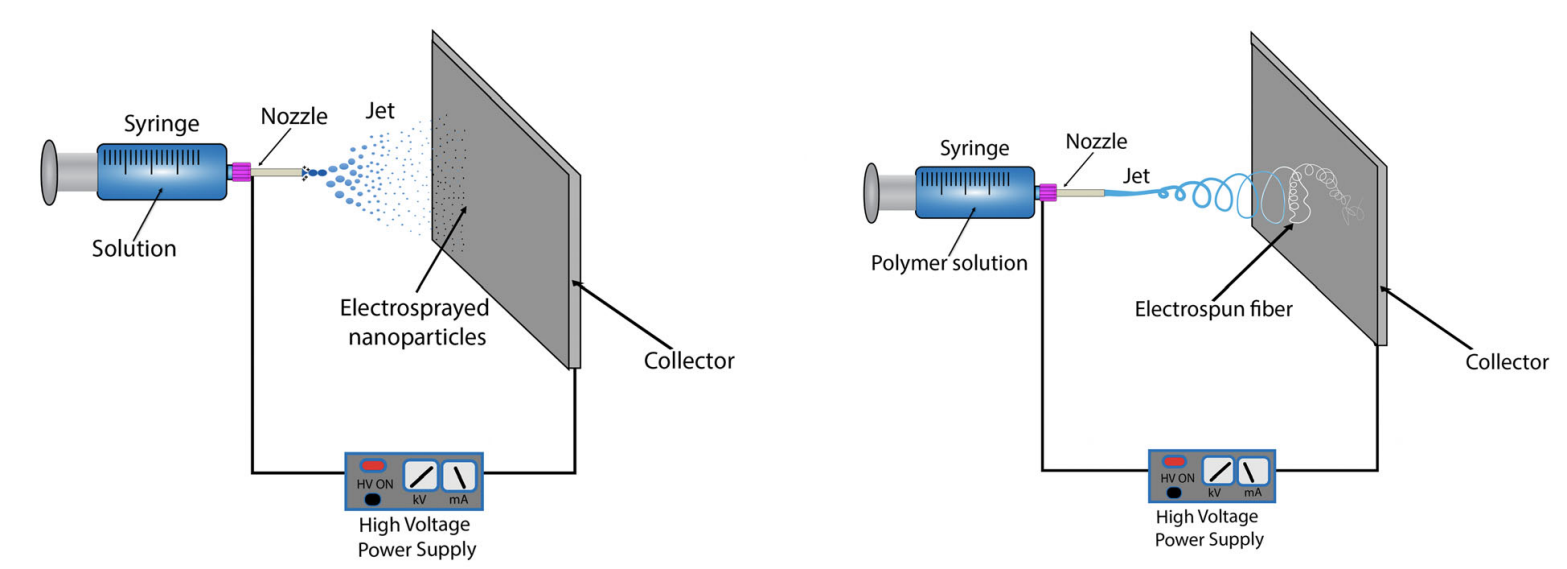
Left: Schematic drawing of a typical electrospray setup. Right: Schematic drawing of a typical electrospinning setup

Both electrospray and electrospinning methods use analogous technology for the production of nanostructures. The different modes are determined by the properties of the applied solution in the process. A typical laboratory electrospinning setup can be capable of both the electrospray and electrospinning modes. In general, the setup is made of 4 main parts (Fig. 1): (i) a syringe, which is placed inside a syringe pump for continuous solution flow; (ii) a metallic nozzle; (iii) a high voltage power supply (which is connected to the nozzle); (iv) and a collector (which is conductive to attract the charged nanoparticles/nanofibers, and is placed opposite to the high voltage electrode) [3, 22].

Depending on the viscosity and electrical conductivity of the solution, the setup can be used in either electrospray or electrospinning modes. In both cases, the liquid that is ejected from the nozzle forms a specific cone geometry, called Taylor cone [23]. In the electrospray mode, highly charged droplets are ejected as a form of a jet from the Taylor cone, and upon solvent evaporation, solid nanoparticles can be collected [3]. While in electrospinning mode, continuous fibers are emitted from the Taylor cone, and the nanofibers solidify after the complete solvent evaporation [24]. Ideally, both drying processes occur before the nanoparticles/nanofibers would reach the collector. Figure 1 shows the differences and similarities between the two modes.

Even though the experimental setup for electrospray and electrospinning methods are fairly simple, the price range of a commercial setup is usually between $17,000 - $300,000 USD [25]. Many researchers all around the world are using unsafe home-built experimental setups, where the users can be exposed to electric shock from the high voltage components. FDM 3D printing offers a low-cost solution to print a setup that offers similar reliability and reproducibility of the results as the commercial ones.

This paper describes the 3D printing process of a safe, modular electrospray/electrospinning setup. The detailed method for 3D printing this device that includes engineered air channels for enhanced solvent evaporation is described in detail. The optimal printing parameters are given, and both the .sldprt and .stl files are provided. The chemical smoothening and assembly of the 3D-printed parts are also described.

## Experimental material and methods

### Materials

Both the polylactic acid (PLA) and polyvinyl alcohol (PVA) filaments (diameter 2.75 mm) were manufactured by Ultimaker B.V., The Netherlands. A polyester-based thermoplastic elastomer filament (diameter 3 mm) was manufactured by Mitsubishi Kagaku Media Co., Ltd., Japan, and marketed under the name ‘Verbatim Primalloy’. All three filaments were purchased from Create Education Limited, UK.

### 3D printer

An Ultimaker 3 FDM 3D printer was purchased from Ultimaker B.V., The Netherlands for printing the parts of the setup. The printer is equipped with a dual extrusion nozzle system. Thus, it is capable of using a second extruder for water-soluble support structure printing. Print cores with the 0.4 mm nozzles were used for both materials.

### CAD design and G-code

The model of the electrospray/electrospinning chamber was designed by SolidWorks software. The SolidWorks Part files (.sldprt file format) can be downloaded at the journal website of the Hypertext Markup Language (HTML) format article. The files were exported as .stl files (can also be downloaded at the website of the HTML format article), and Ultimaker’s slicer software, Cura (ver. 3.20), was used to generate the G-codes. The print settings can be found in Table 1.

**Table 1:** The main print settings for the PLA, PVA and Verbatim Primalloy filaments

PLA filament print settings	
Print temperature	210 °C
Build plate temperature	60 °C
Print speed	20 mm/s
Layer height	0.15 mm
Infill	20%
Build Plate Adhesion	Brim, 3 mm
Prime Tower Size	10 mm
PVA filament print settings	
Printing Temperature	213 °C
Flow	120%
Print Speed	20 mm/s
Verbatim Primalloy filament print settings	
Printing Temperature	230 °C
Build plate temperature	60 °C
Print Speed	20 mm/s

## Results and discussion

**Fig. 2:**
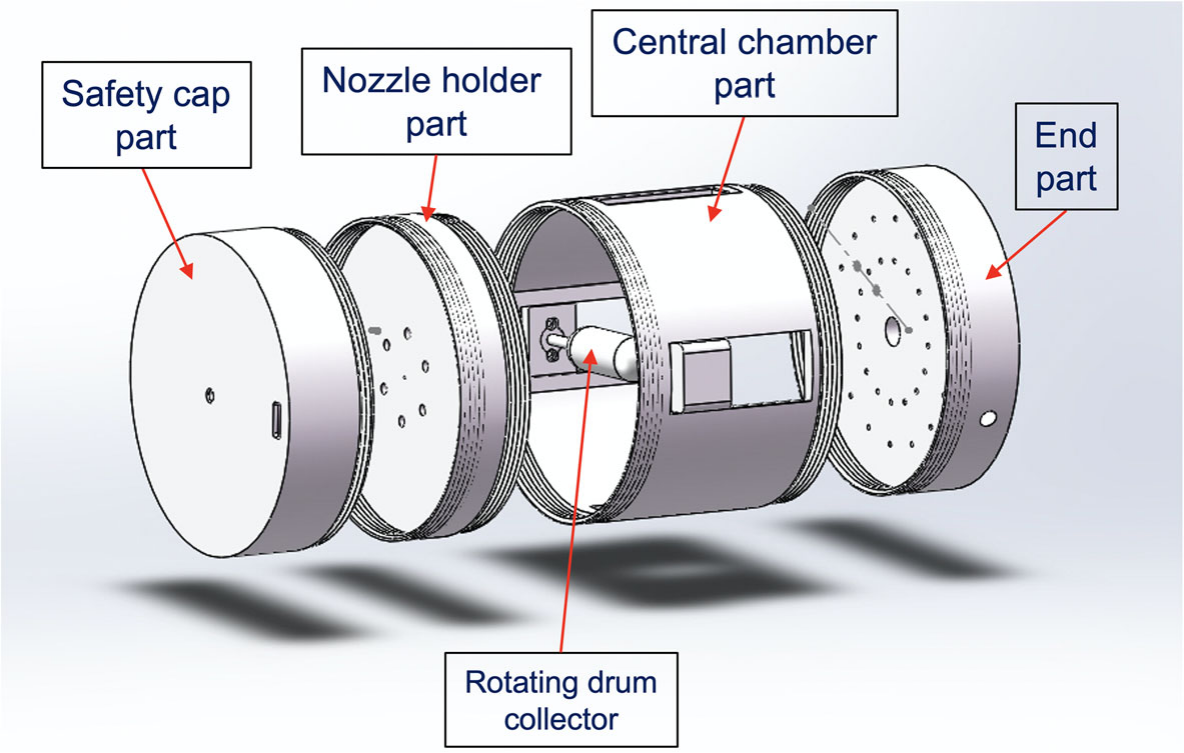
Electrospray/electrospinning chamber CAD drawing with the rotating drum collector present

The electrospray/electrospinning setup was assembled as shown in Fig. 2. The chamber consisted of four main parts: a safety cap, a nozzle holder, a central chamber part, an end part with gas channels and stands to keep the setup in place. Either a stationary rod collector or a rotating drum collector was used to collect the nanoparticles/nanofibers (Fig. 2 shows the rotating drum collector). PVA was used as support material during printing the parts with complex structures, e.g. the safety cap, the larger nozzle holding part, the chamber and the end part. After a part with PVA support was printed, 30 °C water was used to dissolve the PVA in a water bath. It took approximately 24 h to fully dissolve the PVA at this temperature. All the larger parts were printed with a sheet of paper attached to the open front part of the Ultimaker 3 printer, in order to reduce the temperature fluctuations during the 3D printing process. The rig shown in Fig. 2 was printed in 6 days, with the filaments costing $100 USD.

### 3D printing the nozzle holder and the safety cap

**Fig. 3:**
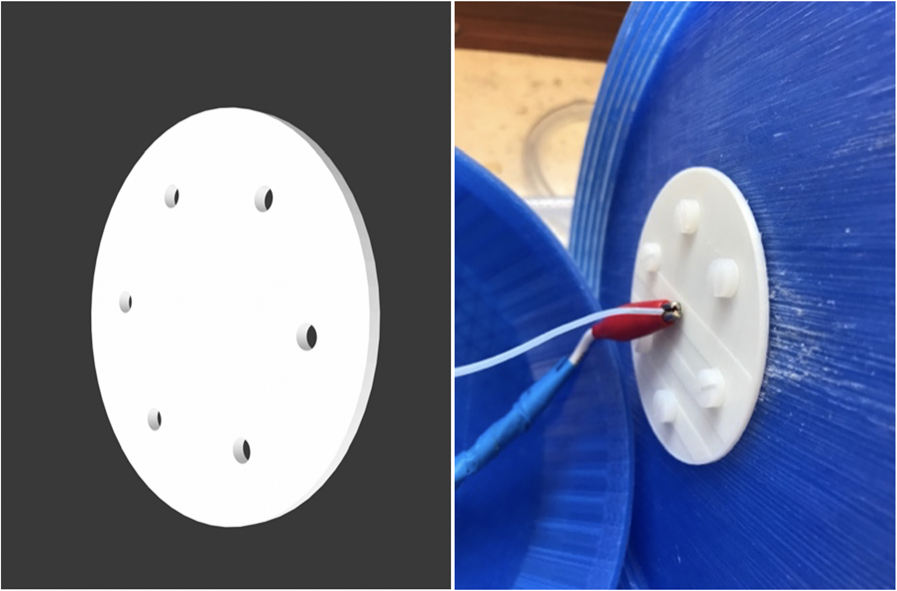
Left: CAD design of the electrospray/electrospinning nozzle capillary holder part. Right: Photograph of the white nozzle capillary holder part that has been printed from a rubber material, marketed under the name Verbatim Primalloy. The high voltage cable of the power supply was clamped on the metallic part of the nozzle with the help of a crocodile clip. The Teflon tube from the syringe pump was slid onto the nozzle that was piercing through the elastic rubber part

The nozzle holder consisted of two parts. The larger part was 3D-printed using PLA, as it is a widely used low-cost thermoplastic. However, due the sensitivity of PLA to solvents, heat and moisture would make other thermoplastic materials, like ABS or PEEK, more suitable for long-term use of the setup. While ABS is only 12% more expensive than PLA [26], the price of PEEK filament is over 17 times higher than that of PVA of the same weight [27]. Furthermore, the used PLA material was not able to hold the metal nozzle capillary securely. Therefore, the electrospray/electrospinning nozzle capillary was held in place by a thermoplastic elastomer material, which is marketed under the name ‘Verbatim Primalloy’. This material has outstanding heat, oil and abrasion resistance as well as superior mechanical strength [28]. The 3D-printed white rubber disk was attached to the nozzle holder part (blue part in Fig. 3) using six M5 nylon screws. The blunt nozzle pierced the rubber disk in the middle and was kept securely. The Teflon tube from the syringe pump and the high voltage cable from the power supply were attached to the nozzle (Fig. 3 Right).

**Fig. 4:**
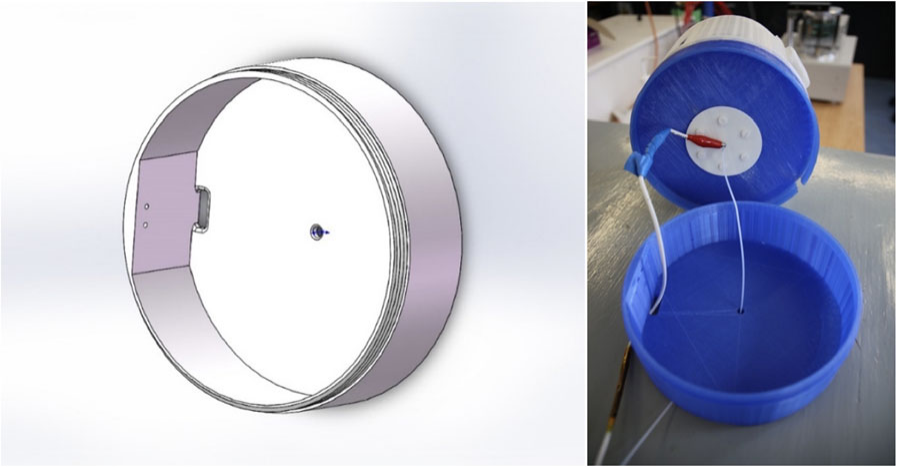
Left: The CAD drawing of the safety cap. Right: Photograph of the safety cap with the high voltage cable on the left slide and solvent feed tube in the center

The safety cap is a crucial element, as it prevents users from electric shock. This part was printed with two small openings: one central hole for the Teflon tube, and one opening for the high voltage cable. The CAD drawing and a photograph of the part can be seen in Fig. 4. This part was connected to the nozzle holder part via threads.

### 3D printing the central chamber parts

**Fig. 5:**
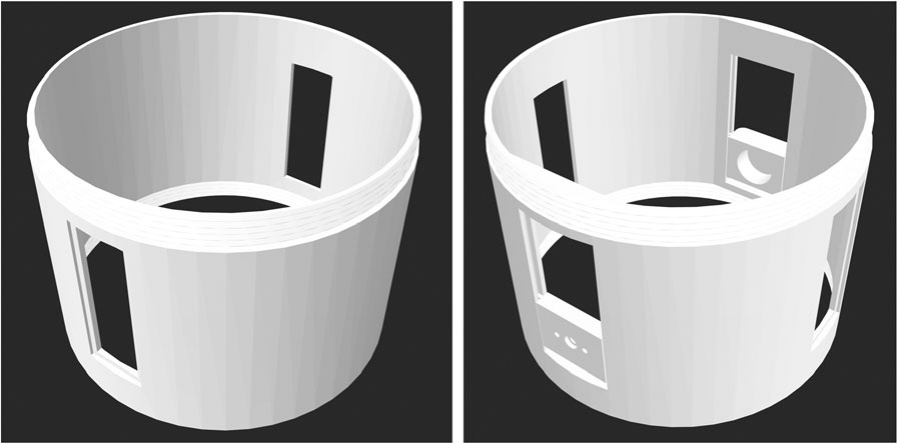
Left: CAD drawing of the chamber part with the viewing windows for use with the stationery collector. Right: CAD drawing of the chamber part with the additional openings for the sliding mechanism. The three holes on the left side of the chamber are for the motor shaft and the two screws holding the DC motor in place. The large hole opposite the three holes is for the ball bearing

Two different designs were made for the central chamber parts. One design was for using the setup with a flat stationary collector, while the second design was for using it with a rotating drum collector (Fig. 5). The two parts were based on the same design, but the part for the rotating collector electrode had two additional openings, where the DC motor and the bearing was able to slide, making the working distance of the collector adjustable.

**Fig. 6:**
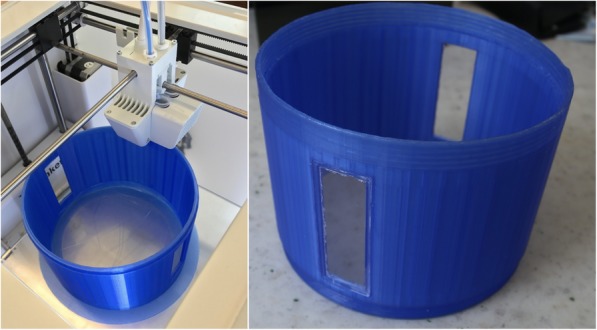
Left: The 3D printing process of the chamber part with the viewing ports. Right: Photograph of the 3D printer during printing the chamber part with the two plexiglass windows glued in place

The central chamber was the largest part of the setup, with 180 mm height and 180 mm diameter. It took 48 h to print this part of the setup. Figure 6 shows the 3D printing process of this part, and the part after completion. Plexiglass was cut to match the size of the opening and glued with transparent silicone in place.

### Rotating drum and sliding stationary flat collector design

**Fig. 7:**
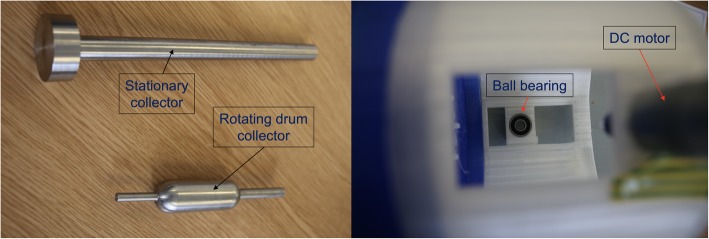
Left: Photograph of the stainless steel stationary flat collector (top) and the rotating drum collector (bottom). Right: Photograph of the ball bearing, which houses the shaft of the rotating drum collector. The blurry part on the right side is the DC motor

In order to provide good electrical conductivity and chemical resistance against solvents, both collector parts were machined from stainless steel, using computer numerical control (CNC). The stationary flat collector consisted of a cylinder part with a diameter of 40 mm (Fig. 7) and a rod. The cylinder part was attached to a 300 mm long rod by threads, and the working distance (distance between the nozzle and the collector surface) was adjusted by sliding the rod part of the collector inside the chamber. The rotating collector drum offers the possibility to collect align fibers, thicker mat than what can be obtained with the stationary collector, and increased production rate [24, 29]. It had a total length of 160.5 mm, with shafts with different diameters on both ends (Fig. 7). The shaft with 6 mm diameter was connected to a 100 rpm DC motor via a metallic coupler. The stainless steel collector was grounded via the DC motor. The shaft with 9 mm diameter was inserted into a metallic ball bearing (Fig. 7 Right) to facilitate the high-speed rotation without extensive friction. The working distance was adjusted by sliding the bearing along with the DC motor. The wider part of the collector drum was 25 mm in diameter, and it provided 50 mm flat surface for collecting the nanoparticles/nanofibers.

### 3D printing the end part with gas channels

**Fig. 8:**
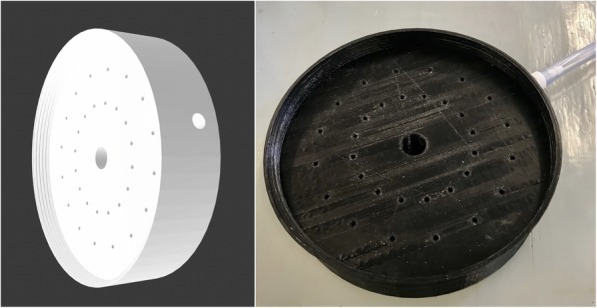
Left: CAD drawing of the end part with the hole for the gas inlet on the side, and a larger hole for the stationary collector rod in the center. Right: Photograph of the end part with the gas tube attached

The electrospray/electrospinning chamber was closed with an ‘end part’, which allowed gas to be entered into the chamber. The cap had a showerhead-like design with multiple holes to diffuse the gas blown into the chamber (Fig. 8). This part had several gas channels, allowing a uniform gas flow. The direction of the gas is opposing the direction of the nanoparticles/nanofibers, enhancing the evaporation rate, thus the drying process.

### Support stands

**Fig. 9:**
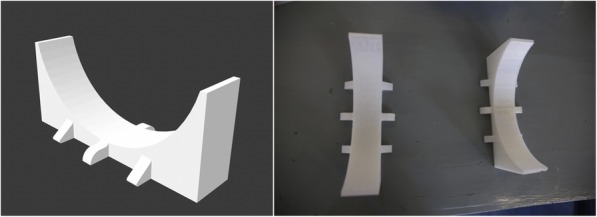
Left: CAD drawing of a support stand. Right: Photograph of the two 3D-printed support stands

The setup was kept in place and prevented from rolling by two 3D-printed support stands (Fig. 9). The stands were printed from PLA, without using any support materials.

### Chemical surface smoothening

In order to smoothen the threads of the modular parts, chloroform vapor was used to treat the 3D prints. It has been previously demonstrated that chloroform can not only reduce the roughness, but also increases the tensile strength of specimens in the upright build direction, increasing the overall material quality [30]. About 50 mL chloroform was poured in a glass beaker inside a fume cupboard, and heated to 250 °C. The beaker size was slightly larger in diameter than the printed part that was treated. The parts were fixed above the beaker, and each side was left for 10 min above the chloroform. The chemical treatment showed a slight improvement in the surface roughness, which helped the part assembly at the threads.

### Assembly and testing

**Fig. 10:**
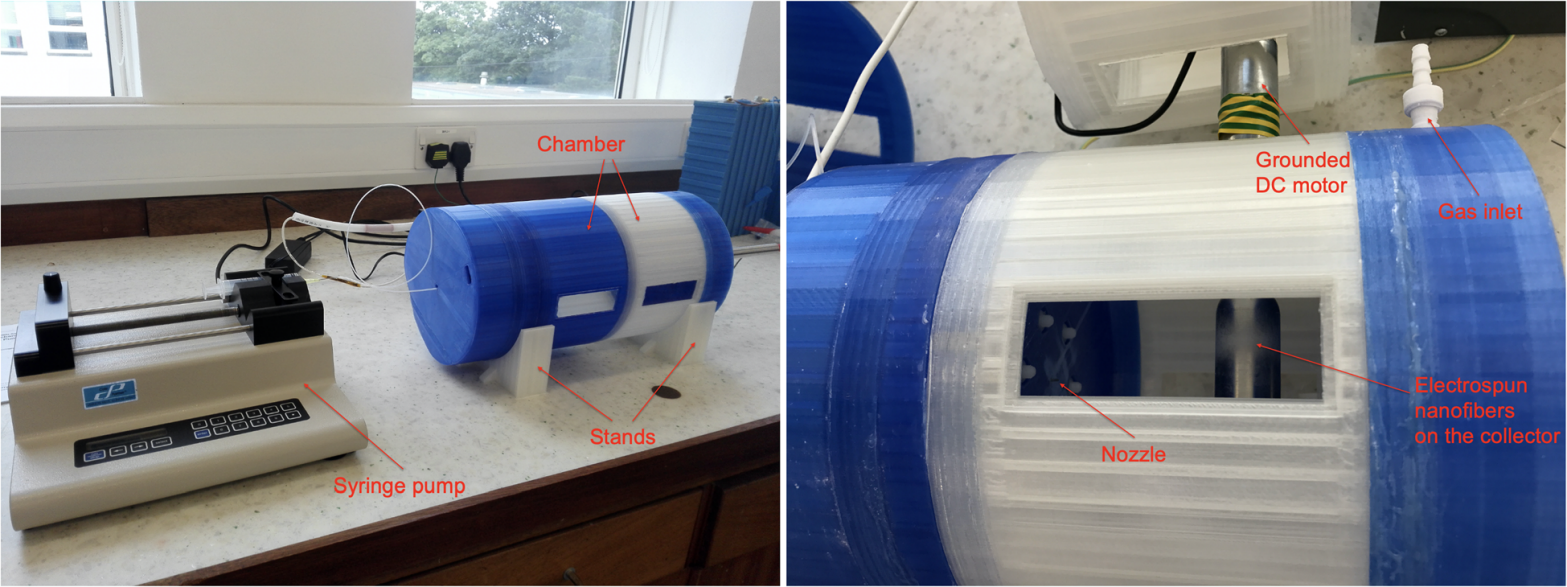
Left: The 3D-printed setup with two chamber parts assembled in electrospray mode. Right: The 3D-printed setup with the rotating collector chamber part during electrospinning nanofibers

The setup was assembled in both electrospray and electrospinning modes. Application of a lubricant on the threads helped the assembly and disassembly process of the parts. Figure 10 shows the assembled setup in electrospray mode, with the syringe pump.

## Conclusion

3D printing offers a low-cost way to manufacture easily a safe and reliable experimental setup that is similar to the commercial ones. This paper presented a method for 3D printing a modular electrospray/electrospinning setup using an inexpensive FDM 3D printer. Both electrospray and electrospinning techniques are widely used for drug delivery, tissue engineering, biosensing, or replica molding applications in the recent years. PLA, PVA and thermoplastic elastomer filaments were used for the 3D printing process, with filaments costing only $100 USD. An Ultimaker 3 printer (with dual print heads) was employed and the PVA was used as water-soluble support. The electrospray/electrospinning rig was printed in less than a week. Due to the modular nature of the setup, the parts can be exchanged easily, offering easy configuration for different applications. The cap part had several gas channels, allowing a uniform gas flowing against the direction of the nanoparticles/nanofibers, enhancing the evaporation rate. The setup was tested in both electrospray and electrospinning modes successfully. However ABS, PEEK, or ceramic materials would be recommended for 3D printing the central chamber part in order to increase the chemical resistivity. Both the .sldprt and .stl files are provided for download.

## Supplementary information

**Additional file 1.** CAD drawings of the safety cap.

**Additional file 2.** CAD drawing of the nozzle holder.

**Additional file 3.** CAD drawing of the cylindrical part with windows.

**Additional file 4.** CAD drawing of the cylindrical part with windows and sliding rotating collector base.

**Additional file 5.** CAD drawing of the support stand part.

**Additional file 6.** CAD drawing of the end part with air channels.

**Additional file 7.** All the STL files of the parts compressed.

## Data Availability

All data generated or analyzed during this study are included in this published article. The CAD and STL files can be downloaded with no costs.

## Abbreviations

3D: Three dimensional
CAD: Computer-aided design
CNC: Computer numerical control
EHDA: Electrohydrodynamic atomization
FDM: Fused deposition modelling
HTML: Hypertext Markup Language
PLA: Polylactic acid
PVA: Polyvinyl alcohol
STL: Standard tessellation language
